# Prognostic Value of Admission Blood Glucose in Diabetic and Non-diabetic Patients with Intracerebral Hemorrhage

**DOI:** 10.1038/srep32342

**Published:** 2016-08-26

**Authors:** Shichao Sun, Yuesong Pan, Xingquan Zhao, Liping Liu, Hao Li, Yan He, Yilong Wang, Yongjun Wang, Li Guo

**Affiliations:** 1 Department of Neurology, The Second Hospital, Hebei Medical University, Shi Jiazhuang, Hebei Province, China; 2 Department of Neurology, Beijing Tiantan Hospital, Capital Medical University, Beijing, China; 3 Department of Epidemiology and Health Statistics, School of Public Health, Capital Medical University, Beijing, China; 4 China National Clinical Research Centre for Neurological Diseases, Beijing, China; 5 Centre of Stroke, Beijing Institute for Brain Disorders, Beijing, China; 6 Beijing Key Laboratory of Translational Medicine for Cerebrovascular Disease, Beijing, China

## Abstract

We aimed to validate prognostic value of elevated admission blood glucose (ABG) for clinical outcomes in diabetic and non-diabetic patients with intracerebral hemorrhage (ICH) in a representative large cohort. Data of ICH patients with onset time ≤24 h were derived from the China National Stroke Registry. Clinical outcomes included 3-month poor outcome (death or dependency) and death. Logistic regression was performed for the association between ABG and clinical outcomes, both in the entire cohort and in patients with and without diabetes mellitus. 2951 ICH patients were enrolled, including 267 (9.0%) diabetics. In the entire cohort, there was a trend to increased risk of poor outcome with increasing ABG levels (adjusted OR 1.09; 95% CI, 1.04–1.15; P < 0.001). The risk of poor outcome was significantly greatest for the highest quartile (≥7.53 mmol/L) of ABG (adjusted OR 1.54; 95% CI, 1.17–2.03; p = 0.002, P for trend 0.004). We got similar association in non-diabetics but not in diabetics. Elevated ABG confers a higher risk of poor outcome in non-diabetics than diabetics with similar glucose level. Elevated ABG is an independent predictor of 3-month poor outcome in ICH patients, the prognostic value of which is greater in non-diabetics than diabetics with similar glucose level.

Intracerebral hemorrhage (ICH) accounts for 10–15% of all stroke cases in Western countries and up to 20% to 30% in Asian countries^1^, which is associated with higher rates of death and disability than ischemic stroke^2^. The evaluation and control of predictors for ICH clinical outcomes is of great importance.

Elevated admission blood glucose (ABG) has been linked to a poor prognosis in patients with ischemic stroke^3^,^4^. However, the prognostic value of elevated ABG for ICH outcomes is still under debate and it is unclear whether elevated ABG portended a different prediction based on patients’ diabetic status. Some^5^,^6^ but not all^7^,^8^ studies have shown that elevated ABG is a predictor of poor outcomes in ICH, most of which are limited to small size, single-centre design, or no direct comparison between diabetics and non-diabetics. Evidences from large-scale multi-centre studies are quite limited^9^,^10^, the study subjects of which are limited to specific population such as non-comatose^9^ or mild to moderate patients^10^. Accordingly, the purpose of this study is to validate the prognostic value of elevated ABG for clinical outcomes in diabetic and non-diabetic patients with intracerebral hemorrhage (ICH) in a representative large cohort.

## Results

### Characteristics at baseline

**Figure 1:**
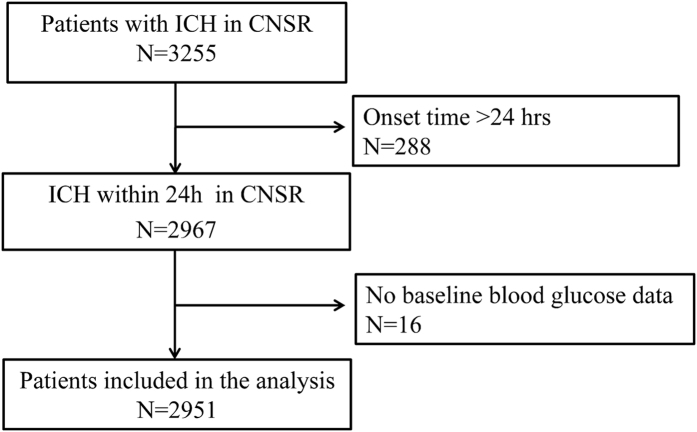
Patient flow diagram.

There were 3255 ICH patients meeting the diagnosis criteria for ICH in our study, of which, 288 (8.8%) were excluded due to onset time >24 hours and 16 (0.5%) were excluded for missing ABG measurements (Fig. 1). Among 2951 remaining patients, 1156 (39.2%) were female and 1795 (60.8%) were male. The ages of the participants ranged from 18 to 98 years and mean ± standard deviation were 62 ± 13 years. The median Glasgow Coma Scale (GCS) score of the participants was 14 (interquartile range, 8 to 15), and the median National Institutes of Health Stroke Scale (NIHSS) score was 9 (interquartile range, 3 to 17). The baseline characteristics of the patients are presented in Table 1.

DM was presented in 267 (9.0%) patients. The median (interquartile range) ABG levels for the entire cohort, diabetics and non-diabetics were 6.4 (5.8–7.5) mmol/L, 7.7 (6.3–10.7) mmol/L, and 6.3 (5.7–7.3) mmol/L, respectively. The distributions of ABG levels in these groups are presented in Supplementary Fig. S1. Elevated ABG was common in non-diabetics (e.g., 22% in the highest quartile, 3.6% in the group with ABG ≥11.1 mmol/L). Compared with patients who had lower ABG, patients with elevated ABG were more likely to be female, have more risk factors, had greater stroke severity, were more likely to be with a history of oral hypoglycemic agents and insulin treatment, tended to be treated with neurosurgical intervention, NICU/ICU care or withdraw of support and stayed longer in hospitals.

### Associations between ABG and outcomes

**Figure 2:**
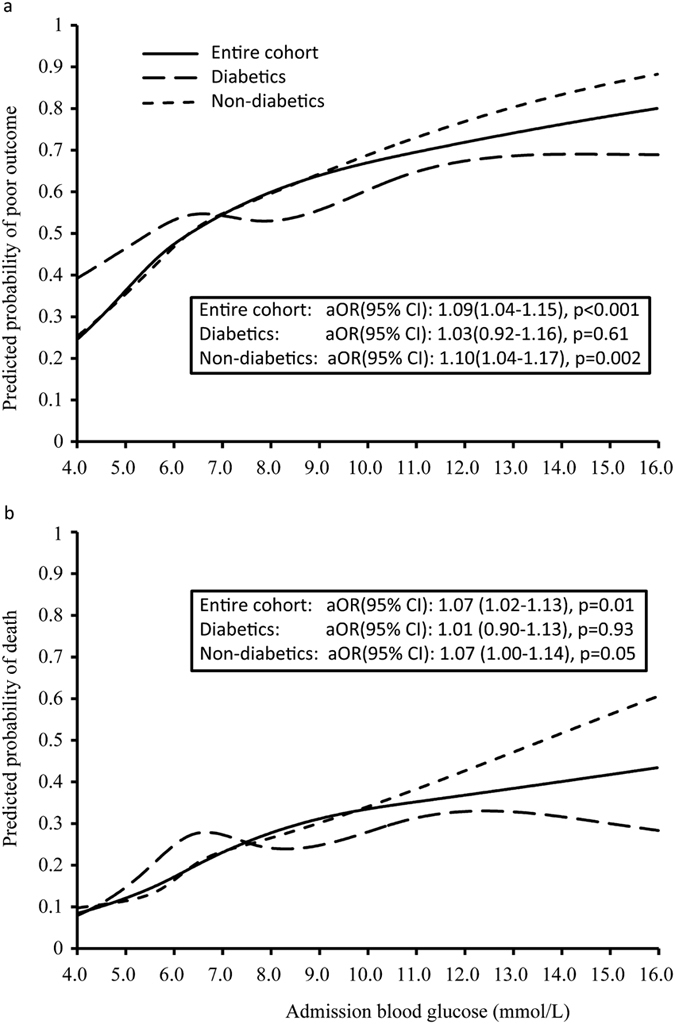
Predicted probabilities of poor outcome (**a**) or death (**b**) by admission blood glucose.

No collinearity or significant interactions was found between baseline variables. When ABG concentration was analyzed as a continuous variable, a trend to increased risk of poor outcome with increasing ABG concentration was presented (Fig. 2a) in the entire cohort (adjusted OR 1.09; 95% CI, 1.04–1.15; P < 0.001) and non-diabetics (adjusted Odds ratio [OR] 1.10; 95% confidence interval [CI], 1.04–1.17; P = 0.002), but not in diabetics (adjusted OR 1.03; 95% CI, 0.92–1.16; P = 0.61). Although non-diabetics at the lower ABG levels had a lower poor outcome risk than diabetics, their risk increased more steeply at higher ABG levels, surpassing the risk of diabetics at about 7.0 mmol/L (Fig. 2a, P for interaction <0.001). Similar phenomenon existed for the risk of death, with the risk in non-diabetics surpassing that of the diabetics at an ABG level of 7.5 mmol/L around (Fig. 2b, P for interaction <0.001).

When ABG levels were stratified by quartiles (Table 2), there was a graded increase in the risk of poor outcome as ABG became progressively elevated. The risk of poor outcome was significantly greatest for the highest quartile (adjusted OR 1.54; 95% CI, 1.17–2.03; P = 0.002, P for trend 0.004). Similar results were evident for death (adjusted OR 2.05, 95% CI, 1.44–2.92, P for trend 0.002). These relationships presented in non-diabetics but not in diabetics (Table 3). Similar results were found when reclassifying glucose levels by tertiles or by diagnostic threshold with ABG ≥11.1 mmol/L (Supplementary Table S1 and Supplementary Table S2). Adjusted common OR (95% CI) for quartile 2, quartile 3 and quartile 4 in ordinal regression model were 1.33 (1.09–1.61), 1.32 (1.09–1.61) and 1.47 (1.20–1.79), respectively.

## Discussion

It is important to determine whether elevated ABG after ICH is associated with increased poor outcome, since this factor may be therapeutically modified. Our study indicates that elevated ABG is an independent predictor of 3-month poor outcome in ICH patients, the prognostic utility of which might be altered by patients’ diabetic status. Poor outcome risk is greater in non-diabetics with elevated ABG than diabetics with similar ABG level.

Our finding of elevated ABG being a predictor of poor outcomes in ICH is opposite to some previous studies^7^,^8^, the small sample size, single-centre design and mixed stroke population of which may account for the discrepancy. Evidence from large-scale multi-centre study^9^,^10^ supports the prognosis value of elevated ABG on poor outcomes in ICH. However, being limited to the specific study population, their findings may not be applicable to patients with severe ICH. Meanwhile, the prognostic utility based on patients’ diabetic status is not clearly elucidated in their study due to no direct comparison between diabetics and non-diabetics. Our China National Stroke Registry (CNSR) cohort study demonstrates that the prognostic utility of elevated ABG could be extended to patients with various severity of ICH and indicates that the prognosis utility is caused by a strong association in non-diabetics. The phenomenon that poor outcome risk is greater in non-diabetics than diabetics with similar ABG level after a specific threshold has been found in acute myocardial infarction^11^. Our study presents this phenomenon in ICH patients as well.

Our study do not shed light on exact pathophysiological mechanisms by which elevated ABG affect prognosis. Previous animal studies indicate that elevated blood glucose may exert deleterious effect on brain through inducing neuronal apoptosis^12^, increasing superoxide production^13^, down-regulating the Aquaporin-4 (AQP-4) expression^14^ and exacerbating perihematomal cell death^15^ in the brain.

Our data show different prognostic utility of elevated ABG in diabetics and non-diabetics. The possible explanations could be as follows. First, non-diabetics may expose to a greater degree of stress to reach the same hyperglycemic state as diabetic counterparts. Previous study has indicated that stress-induced hyperglycemia conferred higher mortality than diabetic hyperglycemia in trauma^16^. Second, insulin resistance may exist in non-diabetic patients with elevated ABG^17^,^18^. Non-diabetics may expose to a greater degree of fluctuation in insulin resistance to reach the same hyperglycemic state as diabetic counterparts, which confers higher risk of adverse outcome^19^. Third, some non-diabetics with elevated ABG (particularly those with glucose ≥ 11.1 mmol/L) are undiagnosed diabetics or pre-diabetics and thus may represent a higher-risk cohort. Forth, elevated ABG in non-diabetics was rarely treated during hospitalization^11^. A better management of elevated ABG in diabetics compared with non-diabetics may result in a relatively better outcome.

Our study validates the associations between elevated ABG and adverse clinical outcomes in ICH patients with various severity and highlight clinical concern on non-diabetic ICH patients with elevated ABG. Whether tight glucose control and different target glucose levels for patients with and without DM can improve the outcome of patients with ICH and elevated ABG needs to be resolved in randomized clinical trials.

Our strengths lie in several aspects. First, our study has a prospective design with representative population and is possibly the largest cohort to date on this issue, which makes our conclusions more reliable. Moreover, associations between ABG and outcomes were compared directly in patients with and without DM to demonstrate different prognostic utility of ABG based on patients’ diabetic status.

Several limitations must be acknowledged. Firstly, the definition of DM status was based only on a medical history at admission, some misclassifications were inevitable. However, the frequency of DM in this study (9.0%) is close to the contemporary prevalence of DM in China^20^ (9.7% in Chinese adults). Secondly, being limited to the spectrum of glucose values in this study, we failed to examine the association between severer levels of ABG and outcomes in patients with DM.

In conclusion, elevated ABG is an independent predictor of 3-month poor outcome in ICH patients, the prognostic value of which is greater in non-diabetic patients than diabetics with similar glucose level.

## Methods

### Study Population

The study was based on the CNSR, a nationwide, multi-centre and prospective cohort study, the design, rationale, and baseline information of which has been described in detail elsewhere^21^. In brief, CNSR was the largest stroke registry of consecutive patients with acute cerebrovascular events between September 2007 and August 2008 in China. 132 hospitals from different regions representing 27 provinces and 4 municipalities in mainland China were selected. CNSR was performed in accordance with the guidelines of the Helsinki Declaration. The protocol and data collection was approved by the Institutional Review Board at Beijing Tiantan Hospital and all participating hospitals. Written informed consent was obtained from each participant or his/her designated relatives.

To be eligible for the diagnosis of ICH in our study, subjects had to meet the following criteria: (1) hospitalized with a primary diagnosis of spontaneous ICH according to the World Health Organization criteria^22^; (2) not including primary intraventricular ICH, ICH caused by trauma, brain tumor, hemorrhage secondary to malignancy, subarachnoid hemorrhage, arteriovenous malformation and hemorrhagic transformation of cerebral infarct; (3) ICH confirmed by brain CT. CT films were collected and assessed for hematoma volume and evaluation of intraventricular extension. All images were prospectively reviewed by a neuroradiologist from each participating center who was blinded to clinical data. The neuroradiologists of the study centres were trained centrally with the CT protocol^23^. Patients were excluded if no admission blood glucose data available. Considering variable intervals between symptom onset and ABG measurement, we excluded patients with onset time over 24 hours as well.

### Data Collection and Variable Definition

Demographic characteristics, clinical information, radiographic findings, treatment during hospitalization and ABG were collected from the database, as well as hemorrhage evaluation including stroke severity, hematoma volume and hematoma location. Stroke severity was measured using the initial NIHSS score and GCS score. ICH hematoma volumes were measured on the initial brain CT by the ABC/2 method^23^. Data on hematoma locations, which were classified as supratentorial and infratentorial, and the presence of intraventricular extension were also collected.

ABG was the random blood glucose^9^,^24^ measured at the initial emergency department or the blood glucose value from in-hospital immediate evaluation, which was generally done within 3 hours of admission. Patients with a history of diabetes or glucose-lowering treatment before ICH were classified as diabetics, according to previously published articles^9^,^10^.

### Outcome Measures

The clinical outcomes was poor outcome defined as death or dependency (modified Rankin scale^25^ [mRS] score of 3 to 6) and death (mRS score of 6) at 3 months, which was assessed by trained study investigators. The telephone follow-up was conducted centrally for all enrolled patients with a standardized interview protocol.

### Statistical Analysis

Baseline demographic and clinical characteristics were expressed as mean (standard deviation) or median (interquartile range) for continuous variables and as number (%) for categorical variables. The chi-square test for categorical variables and Mann–Whitney test for continuous variables were used as needed.

Associations of ABG, both as continuous and categorical (quartile) variables, and risk of poor outcome and death were evaluated by separate univariable and multivariable logistic regression. ORs with 95% CIs were calculated. All significant (P < 0.05) baseline variables in the univariable analysis were included in the multivariable analysis. Collinearity and interaction between variables were also check. We further evaluated the associations between ABG and risk of poor outcome and death using a multivariable logistic regression model with restricted cubic splines for ABG adjusting for all confounding factors. The 5 knots for spline were placed at the 5^th^, 25^th^, 50^th^, 75^th^, 95^th^ percentiles of ABG. These analyses were performed in the entire cohort and repeated in patients with and without DM. Sensitivity analysis including ordinal regression and different categories of ABG (by tertiles and by diagnostic thresholds of blood glucose for DM) were performed to examine the association between ABG and clinical outcomes. A 2-sided P value <0.05 was set as the level for statistical significance. All analyses were performed with SAS software version 9.4 (SAS Institute Inc, Cary, NC, USA).

## Additional Information

**How to cite this article**: Sun, S. *et al.* Prognostic Value of Admission Blood Glucose in Diabetic and Non-diabetic Patients with Intracerebral Hemorrhage. *Sci. Rep.* **6**, 32342; doi: 10.1038/srep32342 (2016).

## Supplementary Material

Supplementary Information

## Figures and Tables

**Table 1:** Baseline characteristics of ICH patients according to ABG quartiles.

Variable	ABG Levels, mmol/L				
	Q1(<5.76)(n = 729)	Q2(5.76–6.35)(n = 743)	Q3(6.36–7.52)(n = 741)	Q4(≥7.53)(n = 738)	P Value
Age (year), mean (SD)	61.2 ± 13.1	62.2 ± 13.4	62.4 ± 12.5	63.5 ± 13.0	0.001
Female, n (%)	241(33.1)	277(37.3)	302(40.8)	336(45.5)	<0.001
Current smoking, n (%)	309(42.4)	271(36.5)	259(35.0)	258(35.0)	0.008
Heavy drink, n (%)	91(12.5)	90(12.1)	81(10.9)	67(9.1)	0.16
Medical history, n (%)					
Diabetes mellitus	18(2.5)	62(8.4)	50(6.8)	137(18.6)	<0.001
Hypertension	256(35.1)	241(32.4)	221(29.8)	210(28.5)	0.03
Dyslipidemia	48(6.6)	62(8.3)	47(6.3)	46(6.2)	0.33
Cardiovascular disease	44(6.0)	64(8.6)	62(8.4)	84(11.4)	0.004
Atrial fibrillation	4(0.6)	13(1.8)	18(2.4)	15(2.0)	0.03
Stroke	169(23.2)	193(26.0)	222(30.0)	220(29.8)	0.008
Pre-disability	118(16.2)	146(19.7)	138(18.6)	165(22.4)	0.03
Stroke severity, median (IQR)					
NIHSS score on admission	7(3–12)	9(3–18)	9(3–16)	13(5–23)	<0.001
GCS score on admission	15(12–15)	14(8–15)	14(9–15)	11(7–15)	<0.001
Hematoma location, n (%)					0.049
Supratentorial	653(89.6)	662(89.1)	650(87.7)	630(85.4)	
Infratentorial	76(10.4)	81(10.9)	91(12.3)	108(14.6)	
Intraventricular extension, n (%)	141(19.3)	199(26.8)	232(31.3)	310(42.0)	<0.001
Hematoma volume (mL)	9.5(4.5–19.5)	12.5(5.6–28.8)	13.2(6.0–28.1)	18.0(7.5–42.0)	<0.001
Supratentorial, n (%)					<0.001
≤30 mL	609(93.3)	545(82.3)	539(82.9)	447(71.0)	
30–60 mL	29(4.4)	74(11.2)	71(10.9)	92(14.6)	
>60 mL	15(2.3)	43(6.5)	40(6.2)	91(14.4)	
Infratentorial, n (%)					0.18
≤10 mL	56(73.7)	55(67.9)	75(82.4)	70(64.8)	
10–20 mL	11(14.5)	15(18.5)	8(8.8)	19(17.6)	
>20 mL	9(11.8)	11(13.6)	8(8.8)	19(17.6)	
Medication history, n (%)					
Oral hypoglycemic agents	10(1.4)	35(4.7)	29(3.9)	74(10.0)	<0.001
Insulin	2(0.3)	14(1.9)	5(0.7)	25(3.4)	<0.001
Antihypertensive agents	285(39.1)	322(43.3)	323(43.6)	331(44.9)	0.13
Antiplatelet agents	56(7.7)	72(9.7)	61(8.2)	79(10.7)	0.17
Anticoagulants	7(1.0)	7(0.9)	8(1.1)	9(1.2)	0.95
Antihyperlipidemia agents	4(0.6)	13(1.8)	4(0.5)	10(1.4)	0.05
Treated in, n (%)					<0.001
Neurology ward/Ward	476(65.3)	499(67.2)	455(61.4)	390(52.9)	
Stroke unit	149(20.4)	96(12.9)	118(15.9)	133(18.0)	
Neurosurgical/Intervention Ward	14(1.9)	21(2.8)	20(2.7)	30(4.1)	
NICU/ICU	90(12.4)	127(17.1)	148(20.0)	185(25.1)	
Medical treatment during hospitalization, n (%)					
Oral hypoglycemic agents	6(0.8)	29(3.9)	33(4.5)	96(13.0)	<0.001
Insulin administration	34(4.7)	53(7.1)	45(6.1)	107(14.5)	<0.001
Antihypertensive therapy	430(59.0)	430(57.9)	432(58.3)	441(59.8)	0.89
Intravenous mannitol	628(86.2)	643(86.5)	650(87.7)	619(83.9)	0.005
Neurosurgical intervention	9(1.2)	14(1.9)	21(2.8)	30(4.1)	0.003
Withdraw of support, n (%)	61(8.4)	110(14.8)	85(11.5)	105(14.2)	0.001
Length of stay (day)	18(13–25)	17(10–25)	19(11–27)	17(7–26)	0.002

ABG indicates admission blood glucose; ICH, intracerebral hemorrhage; Q1, quartile 1; Q2, quartile 2; Q3, quartile 3; Q4, quartile 4; SD, standard deviation; IQR, interquartile range; NIHSS, National Institutes of Health Stroke Scale; GCS, Glasgow Coma Scale.

**Table 2:** Associations between ABG and outcomes.

Outcome	ABG, mmol/L	Events, n (%)	Unadjusted			Adjusted^a^		
			OR (95% CI)	P Value	P for Trend	OR (95% CI)	P Value	P for Trend
Poor outcome	<5.76	253 (34.7)	1.0		<0.001	1.0		0.004
	5.76–6.35	371 (49.9)	1.88 (1.52–2.31)	<0.001		1.31 (1.00–1.71)	0.051	
	6.36–7.52	384 (51.8)	2.02 (1.64–2.50)	<0.001		1.43 (1.10–1.86)	0.01	
	≥7.53	484 (65.6)	3.59 (2.89–4.45)	<0.001		1.54 (1.17–2.03)	0.002	
Death	<5.76	66 (9.1)	1.0		<0.001	1.0		0.002
	5.76–6.35	166 (22.3)	2.89 (2.13–3.93)	<0.001		1.85 (1.29–2.65)	0.001	
	6.36–7.52	146 (19.7)	2.46 (1.81–3.36)	<0.001		1.66 (1.15–2.39)	0.01	
	≥7.53	239 (32.4)	4.81 (3.58–6.47)	<0.001		2.05 (1.44–2.92)	<0.001	

ABG indicates admission blood glucose; OR, odds ratio; and CI, confidence interval. ^a^Adjusted for age, gender, history of hypertension, history of cardiovascular disease, history of atrial fibrillation, history of smoking, baseline hematoma volume and location, intraventricular extension, premorbid modified Rankin Scale score, National Institute of Health stroke scale (NIHSS) score, Glasgow Coma Scale (GCS) score, admitted department, in-hospital treatment of dehydrant agents, craniotomy and withdraw of support.

**Table 3:** Associations between ABG and outcomes.

Outcome	ABG, mmol/L	Events, n (%)	Adjusted^a^ OR (95% CI)	P Value
Poor outcome				
Non-diabetics	<5.76	246 (34.6)	1.0	
	5.76–6.35	332 (48.8)	1.24 (0.94–1.63)	0.13
	6.36–7.52	363 (52.5)	1.47 (1.12–1.93)	0.005
	≥7.53	398 (66.2)	1.51 (1.12–2.02)	0.006
Diabetics	<5.76	7 (38.9)	1.0	
	5.76–6.35	39 (62.9)	2.86 (0.69–11.84)	0.24
	6.36–7.52	21 (42.0)	1.11 (0.25–4.95)	0.15
	≥7.53	86 (62.8)	1.69 (0.46–6.23)	0.89
Death				
Non-diabetics	<5.76	64 (9.0)	1.0	
	5.76–6.35	147 (21.6)	1.81 (1.25–2.64)	0.002
	6.36–7.52	136 (19.7)	1.64 (1.12–2.39)	0.01
	≥7.53	200 (33.3)	1.97 (1.36–2.87)	0.0004
Diabetics	<5.76	2 (11.1)	1.0	
	5.76–6.35	19 (30.7)	1.50 (0.26–8.76)	0.14
	6.36–7.52	10 (20.0)	1.18 (0.19–7.24)	0.65
	≥7.53	39 (28.5)	1.32 (0.25–7.11)	0.86

ABG indicates admission blood glucose; OR, odds ratio; and CI, confidence interval. ^a^Adjusted for age, gender, history of hypertension, history of cardiovascular disease, history of atrial fibrillation, history of smoking, baseline hematoma volume and location, intraventricular extension, premorbid modified Rankin Scale score, National Institute of Health stroke scale (NIHSS) score, Glasgow Coma Scale (GCS) score, admitted department, in-hospital treatment of dehydrant agents, craniotomy and withdraw of support.
