# Supplementary material for: Prognostic Value of Admission Blood Glucose in Diabetic and Non-diabetic Patients with Intracerebral Hemorrhage
Source: Sci Rep. 2016 Aug 26;6:32342. doi: 10.1038/srep32342 (PMC4999808; doi:10.1038/srep32342)
Supplement: Supplementary Information [file srep32342-s1.pdf]

Supplementary Information

Prognostic Value of Admission Blood Glucose in Diabetic and Non-diabetic Patients with Intracerebral Hemorrhage

Shichao Sun, MD; Yuesong Pan, MD; Xingquan Zhao, MD; Liping Liu, MD; Hao Li, PhD, Yan He, PhD; Yilong Wang, MD; Yongjun Wang, MD & Li Guo, MD

Supplementary Table S1. Associations between ABG (by tertiles) and clinical outcomes at 3 months

Outcome	ABG, mmol/L	n	Events, n (%)	Adjusted OR(95% CI)[a]	P Value	P for Trend
Poor outcome	<6.0	972	381 (39.2)	1.0		0.02
	6.0-6.8	980	492 (50.2)	1.24(0.99-1.57)	0.07	
	≥6.9	999	619 (62.0)	1.35(1.07-1.71)	0.01	
Death	<6.0	972	127 (13.1)	1.0		0.06
	6.0-6.8	980	208 (21.2)	1.51(1.12-2.03)	0.007	
	≥6.9	999	282 (28.2)	1.45(1.09-1.94)	0.01	

ABG indicates admission blood glucose; OR, odds ratio; CI, confidence interval.
[a]Adjusted for age, gender, history of hypertension, history of cardiovascular disease, history of atrial fibrillation, history of smoking, baseline hematoma volume and location, intraventricular extension, premorbid modified Rankin Scale score, National Institute of Health stroke scale score, Glasgow Coma Scale score, admitted department, in-hospital treatment of dehydrant agents, craniotomy and withdraw of support.

Supplementary Table S2. Associations between ABG (by diagnostic threshold) and clinical outcomes at 3 months

Outcome	ABG, mmol/L	n	Events, n (%)	Adjusted OR(95% CI)[a]	P Value
Poor outcome	<11.1	2792	1377 (49.3)	1.0	
	≥11.1	159	115 (72.3)	1.69(1.08-2.66)	0.02
Death	<11.1	2792	557 (20.0)	1.0	
	≥11.1	159	60 (37.7)	1.44(0.93-2.22)	0.10

ABG indicates admission blood glucose; OR, odds ratio; CI, confidence interval.

[a]Adjusted for age, gender, history of hypertension, history of cardiovascular disease, history of atrial fibrillation, history of smoking, baseline hematoma volume and location, intraventricular extension, premorbid modified Rankin Scale score, National Institute of Health stroke scale score, Glasgow Coma Scale score, admitted department, in-hospital treatment of dehydrant agents, craniotomy and withdraw of support.

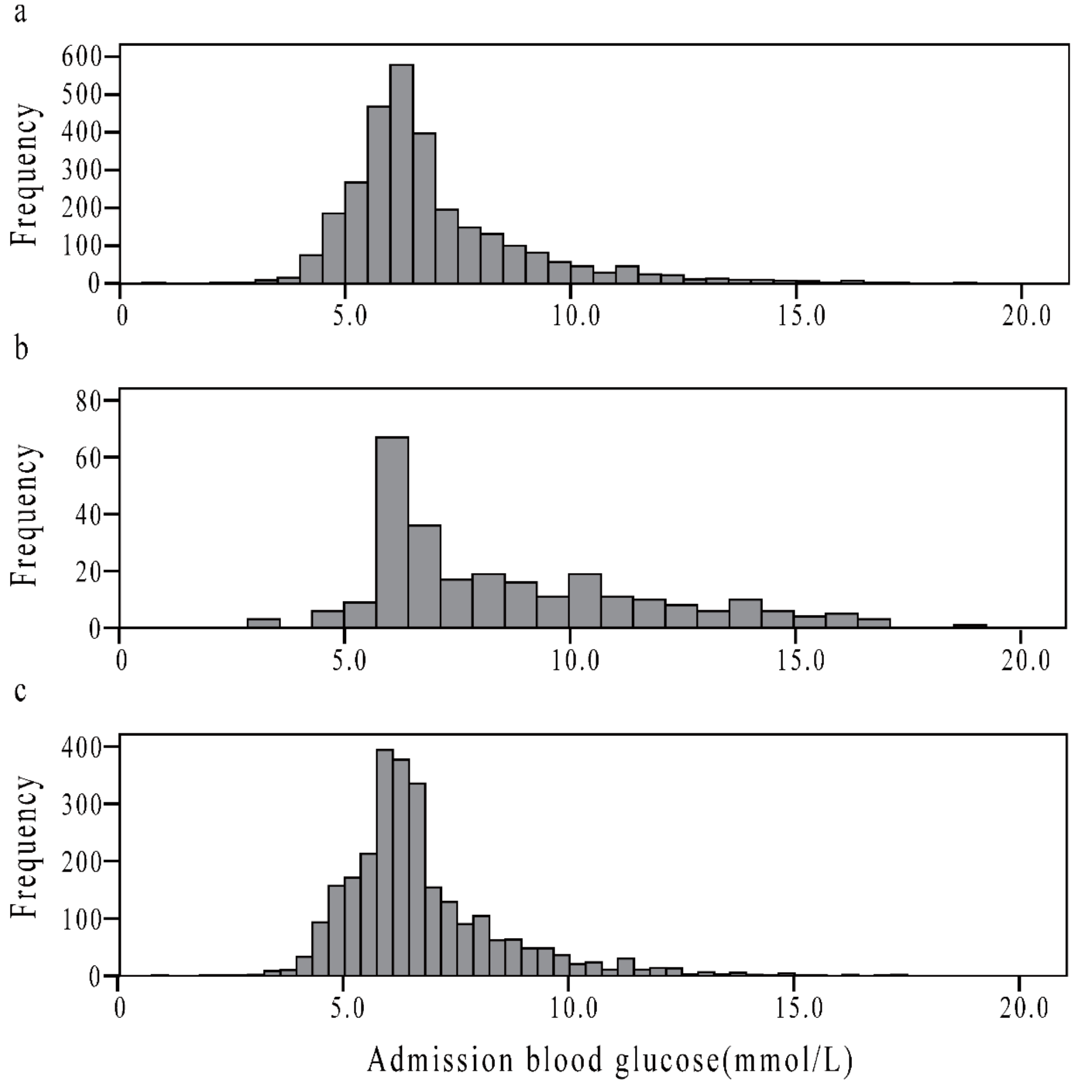

Supplementary Figure S1. Distribution of admission blood glucose in the entire cohort (a), diabetics (b) and non-diabetics (c).
